# Network Patterns of Herbal Combinations in Traditional Chinese Clinical Prescriptions

**DOI:** 10.3389/fphar.2020.590824

**Published:** 2021-01-20

**Authors:** Ning Wang, Ninglin Du, Yonghong Peng, Kuo Yang, Zixin Shu, Kai Chang, Di Wu, Jian Yu, Caiyan Jia, Yana Zhou, Xiaodong Li, Baoyan Liu, Zhuye Gao, Runshun Zhang, Xuezhong Zhou

**Affiliations:** ^1^ Medical Intelligence Institute, School of Computer and Information Technology, Beijing Jiaotong University, Beijing, China; ^2^ Department of Computing and Mathematics, Manchester Metropolitan University, Manchester, United Kingdom; ^3^ Adams School of Dentistry, University of North Carolina, Chapel Hill, NC, United States; ^4^ Department of Biostatistics, UNC Gillings School of Global Public Health, University of North Carolina, Chapel Hill, NC, United States; ^5^ Hubei Provincial Hospital of Traditional Chinese Medicine, Wuhan, China; ^6^ China Academy of Chinese Medical Sciences, Beijing, China; ^7^ National Clinical Research Center for Chinese Medicine Cardiology, Beijing, China; ^8^ Xiyuan Hospital, China Academy of Chinese Medical Sciences, Beijing, China; ^9^ Guanganmen Hospital, China Academy of Chinese Medical Sciences, Beijing, China

**Keywords:** network pharmacology, complex network, herb combination network, clinical prescription, network pattern

## Abstract

As a well-established multidrug combinations schema, traditional Chinese medicine (herbal prescription) has been used for thousands of years in real-world clinical settings. This paper uses a complex network approach to investigate the regularities underlying multidrug combinations in herbal prescriptions. Using five collected large-scale real-world clinical herbal prescription datasets, we construct five weighted herbal combination networks with herb as nodes and herbal combinational use in herbal prescription as links. We found that the weight distribution of herbal combinations displays a clear power law, which means that most herb pairs were used in low frequency and some herb pairs were used in very high frequency. Furthermore, we found that it displays a clear linear negative correlation between the clustering coefficients and the degree of nodes in the herbal combination network (HCNet). This indicates that hierarchical properties exist in the HCNet. Finally, we investigate the molecular network interaction patterns between herb related target modules (i.e., subnetworks) in herbal prescriptions using a network-based approach and further explore the correlation between the distribution of herb combinations and prescriptions. We found that the more the hierarchical prescription, the better the corresponding effect. The results also reflected a well-recognized principle called “*Jun-Chen-Zuo-Shi*” in TCM formula theories. This also gives references for multidrug combination development in the field of network pharmacology and provides the guideline for the clinical use of combination therapy for chronic diseases.

## Introduction

Human disease phenomenon is complicated due to its roots from the disturbance of diverse pathogens on the hierarchical organization of the human life system interacting with the complex natural and social environment. Therefore, finding effective clinical treatments for chronic complex diseases such as diabetes, heart disease, stroke, and cancer is extremely difficult. Furthermore, due to the individualized phenotypes and genotypes incorporated in distinct persons, adverse drug reactions (ADRs) or drug side effects have increasingly become a major health issue when using targeted drugs for patients in real-world clinical settings (Duke et al., 2011; Smyth et al., 2012; Aagaard and Hansen, 2013). For example, there are 106,000 deaths and 2.2 million serious events caused by ADRs in the United States each year (Lazarou et al., 1998), which were once considered as a leading cause of death in the United States (Lazarou et al., 1998). For elderly patients, the ADR is even serious due to the need for multiple prescribed drugs for chronic disease comorbidities (Routledge et al., 2004). However, this problem cannot be well addressed since, for modern drug discovery, the safety of new agents only can be known with certainty if a drug has been on the market for many years. Drugs were only used for selected populations and in limited periods (Lasser et al., 2002) before knowing their safety with great certainty.

To improve the effectiveness of treatment for patients with chronic diseases and comorbidities and the prevention of the emergence of resistance to individual drugs, combination therapy is a new trend in real-world clinical research (Luzuriaga et al., 1997; Mottonen et al., 1999; Wald et al., 2009; Ascierto and Marincola, 2011; Yardley, 2013), which has been effective in the treatment of HIV as well as certain forms of leukemia. Simultaneous combination therapy like two-drug therapy and triple therapy holds huge promise for cancer treatment from the point of cancer cell evolutionary dynamics (Bozic et al., 2013). However, the mechanism and principle underlying effective combination therapy is still a mystery waiting to be investigated (Pritchard et al., 2013).

As a kind of classical combination therapy, traditional Chinese medicine (TCM), which includes multiple herbal ingredients, has been used by Chinese practitioners for thousands of years in real-world clinical settings. Pioneer research (Wang et al., 2008) has already shown that TCM clinical herbal prescription, called formula, consists of both principal and adjuvant components to yield synergy for disease treatment. This demonstrates the molecular mechanism of a well-recognized principle called “*Jun-Chen-Zuo-Shi*” (JCZS) (He et al., 2015; Duan et al., 2018) in TCM formula theories, which illustrates that a well-organized formula should consist of multiple herbal ingredients with differentiated roles like principal role and adjuvant role. Some adjuvant components should be considered to facilitate the delivery of the principal element to the disease site in the body. In real-world clinical herbal prescriptions, some of these have a good therapeutic effect, while others have a poor therapeutic effect. However, the reason why some prescriptions are more effective than others remains to be studied, and there is still a lack of appropriate method to evaluate whether many herbs grouped in certain formulae are suitable for the treatment of specific disease conditions. Therefore, investigation of herbal combination regularities in herbal prescription will benefit the pharmacological understanding of TCM treatment and discovery of novel combination drugs (Li et al., 2010b). In this study, we proposed a network-based approach to investigate the clinical principle of herbal prescribing and quantify the molecular network interactions between herb target modules of a given clinical prescription in the context of the human protein-protein interaction (PPI) network. In addition, we further investigate the correlation between the organization degree of herbal combinations in clinical prescription and their underlying molecular network interaction closeness, which might partially elucidate the organization principle of JCZS in TCM formula theories.

## Materials and Methods

### Herbal Prescription Dataset

To investigate the regularities of herbal combinations in TCM clinical herbal prescriptions, we collected five different datasets from real-world TCM clinical encounters. These five datasets are described in brief in Table 1.

**TABLE 1:** The outline information of five different data sets (in columns) used for analysis.

	Outpatient	Diabetes	CHD	Blood stasis	Qi deficiency
Total number of formulae	531,284	21,626	9,054	2,802	2,393
Distinct number of herbs	576	492	436	439	422
Average frequency of herbs	14,230.02	551.57	270.51	70.65	59.98
Average number of herbs in one formula	15.43	12.55	13.03	11.07	10.58

Outpatient formula data was extracted from the outpatient encounters with one and a half years in one well-recognized general TC hospitals in Beijing, China. The data includes 531,284 different clinical formulae that were used by hundreds of TCM physicians for the management of various diseases (e.g., diabetes, CHD (coronary heart disease), stroke, cancer, chronic gastritis, insomnia, and menopause syndrome) in outpatient encounters. The other four datasets are from inpatient clinical cases, which particularly were prescribed for the patients with type 2 diabetes, CHD, qi deficiency, and blood stasis, respectively. The above-mentioned two diseases (i.e., type 2 diabetes and CHD) are major chronic diseases (He et al., 2019) treated by TCM in real-world clinical settings. The other two syndromes, namely, qi deficiency and blood stasis, are two popular TCM diagnoses for various diseases (Wang et al., 2017). These four inpatient datasets are from a TCM clinical data warehouse (Zhou et al., 2010), which collected structured electronic medical record data from real-world clinical settings in about ten top-level TCM hospitals or wards mostly located in Beijing, China. We calculated the distribution of the number of herbs in one formula for each of the five datasets that represent five clinical prescriptions. The average number in each of the five clinical prescription datasets is 13.43 (outpatient), 9.66 (qi deficiency), 11.6 (diabetes), 12.03 (CHD), and 10.13 (blood stasis). We see that most clinical herbal prescriptions have 5–20 different herbs (over 95% of the five clinical formula datasets have 5–20 herbs) and have more than ten herbs averagely, which showed that clinical herbal prescription is a kind of particular multi-ingredients or drug therapy.

### Construction of Herbal Combination Network

**FIGURE 1:**
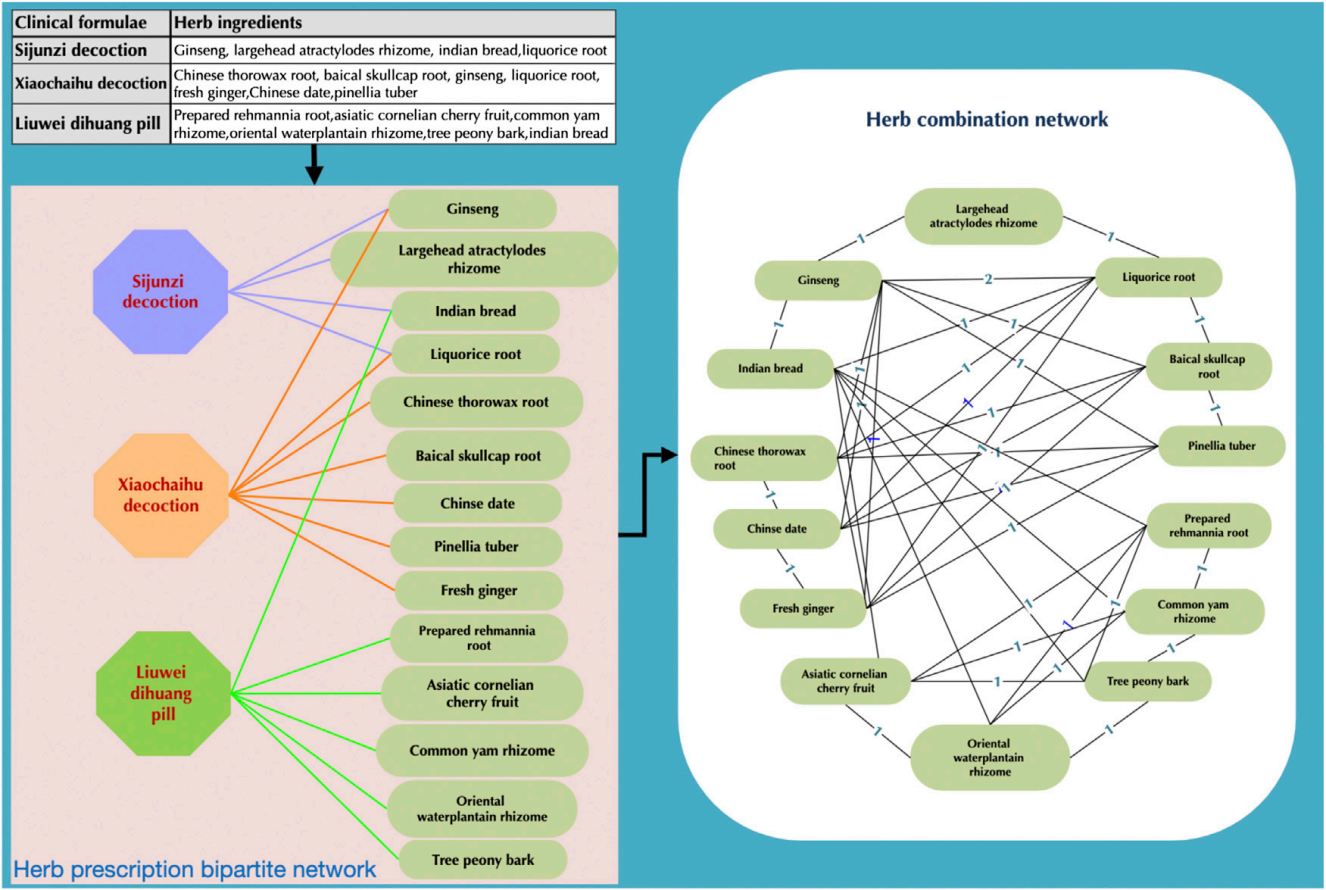
Construction of the bipartite network of TCM clinical formulae. (Table in the left) Three records of clinical formulae, for instance, which include their corresponding herbs. (Left graph) The corresponding bipartite graph of these three clinical formulae, where octagons and oval rectangles correspond to clinical formulae and herbs, respectively. (Right) The herbal combination network where two herbs are connected if they are involved in the same clinical formula. Moreover, the link weight of two herbs corresponds to the number of clinical formulae cooccurring in both herbs.

Considering a clinical formula with multiple herbal ingredients, we can construct a bipartile graph (Figure 1) which has clinical formulae and herbs as two types of nodes. The herbal combination network was constructed by considering herb pairs used in one specific clinical formula if they have been prescribed in more than one clinical formula. Then we would generate links (edges) between these herb pairs. This also means that we would generate a complete graph with each herb in one clinical formula. While a large number of clinical formulae are considered, we can generate a herbal combination network (HCNet, see Figure 1) with nodes representing herbs (herbal ingredients) and edges with a weight representing prescribing cooccurrence in clinical formulae. If two herb pairs are used more frequently than other herb pairs, they would be having a higher weight than other pairs. Furthermore, to compare the patterns of real-world data to those of randomly generated data, we generated the corresponding simulation data with random permutation by using Fisher shuffling (Fisher and Yates, 1948) to get random herb ingredients from herb dictionary to a clinical formula.

### Network Topological Measurements

To investigate the topological properties of HCNet, we use node degree, clustering coefficient, and link weight distribution measurements, which have been widely used in complex network-related studies. The degree of a node is the number of edges incident to the node, which also means the number of herbs combined with an herb in the HCNet. Clustering coefficient evaluates the link density of the neighborhood of a node, which would be near one if most of the nodes were linked in the neighborhood of a node. We used local clustering coefficient of the undirected network to calculate for each node in HCNet, which is calculated as follows:

$$C_{i}=\frac{2\left|e_{jk}:v_{j},v_{k}\in N_{i},e_{jk}\in E\right|}{k_{i}\left(k_{i}-1\right)}$$ (1)

in which $N_{i}$ is the node set of the neighborhood of a node $v_{i}$ in a graph  G(V,E) and $k_{i}$ is the degree of the node $v_{i}$. As a weighted network, HCNet has weight $w_{ij}$ between nodes $v_{i}$ and $v_{j}$, which represents the combination occurrence of herb pairs. We use link weight distribution P(w), which denotes the possibility of a link to have a weight w, to investigate the herbal combination behaviors in HCNet and a given single clinical formula.

### Network-Based Measures of Molecular Interactions Among Herbs in Prescriptions

**FIGURE 2:**
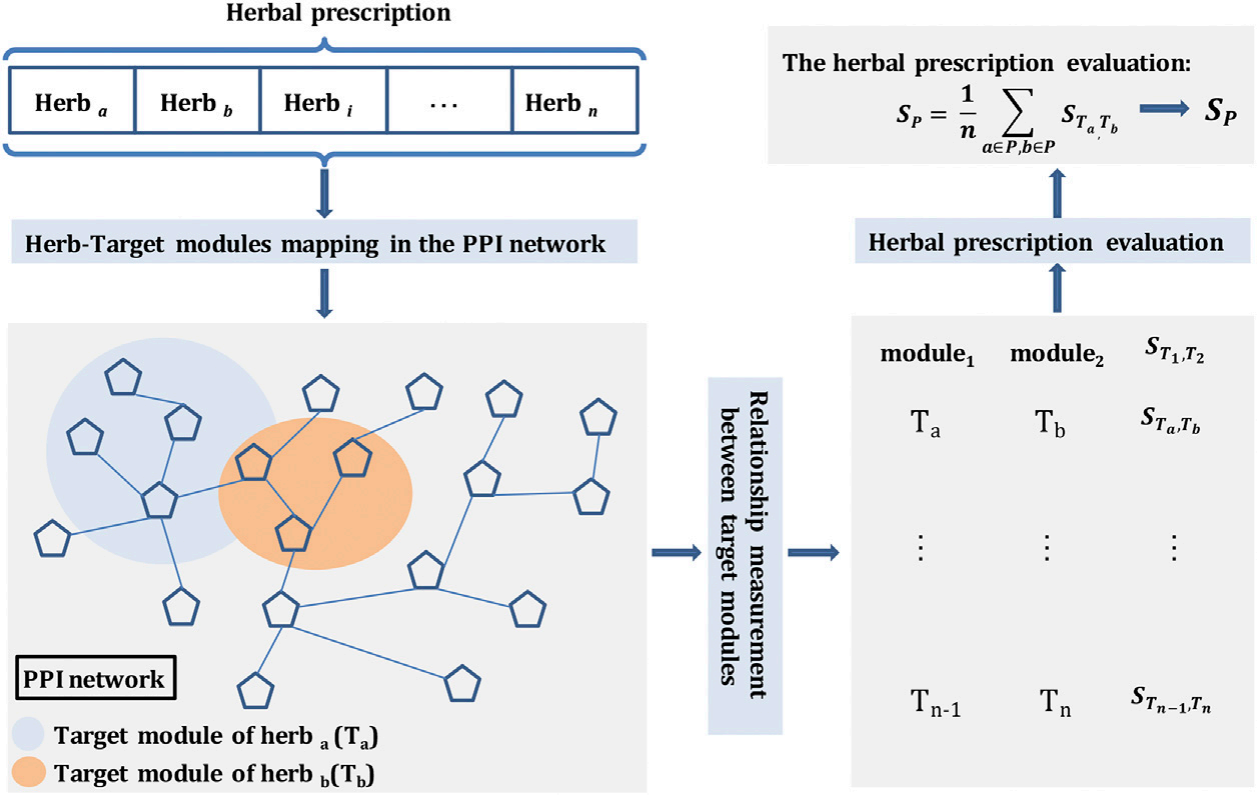
Network-based evaluation measure of herbal prescriptions. A herbal prescription consists of multiple herbs, and herb target modules can be found in the PPI network based on herb-target interactions. Then the relationships between two herb target modules can be measured using the introduced separation measure. Finally, the prescription can be quantified using the relationship of any two herb target modules in the prescription.

As a kind of classical combination therapy, the herbal prescription consists of multiple herbs to treat clinical diseases. Therefore, we adopted a molecular network-based measurement (Menche et al., 2015; Cheng et al., 2019) to quantify the degree of molecular interactions of herbal combinations in each prescription through calculating the average shortest path length between herb target modules (molecular subnetwork of targets) in the context of human PPI network (Figure 2).

Firstly, we measured the network relationship of herb target modules $\;T_{a}$ and $\;T_{b}\;$ in the prescription using the recently introduced separation measure (Menche et al., 2015) to reflect their target localization (Eq. 2):

$$S_{T_{a},T_{b}}=\langle d_{T_{a},T_{b}}\rangle -\;\frac{\langle d_{T_{a},T_{a}}\rangle \;+\;\langle d_{T_{b},T_{b}}\rangle }{2}\;$$ (2)

in which $\;T_{a}$ and $\;T_{b}\;$ represent the target modules of herb a and herb b, respectively. $S_{T_{a},T_{b}}$ compares the mean shortest distances between proteins within each herb, $d_{T_{a},T_{a}}$ and $d_{T_{b},T_{b}}$, to the shortest distances $d_{T_{a},T_{b}}$ between herb a and herb b target pairs. If proteins are associated with both a and b, $d_{T_{a},T_{b}}=0$. For $S_{T_{a},T_{b}}<0$, the targets of the herb a and herb b are located in the same network neighborhood, while for $S_{T_{a},T_{b}}\geq 0$, the two herb targets are topologically separated.

Then, we quantified the herbal prescription using the average of S value between any two herb target modules in the prescription (Eq. 3):

$$S_{P}=\;\frac{1}{n}\;\sum_{a\in P,b\in P}S_{T_{a},T_{b}}$$ (3)

in which $S_{P}$ represents the herbal prescription score and n is the number of herbs in the prescription.

### Hierarchical Organization Measure of Clinical Prescriptions

To further explore the associations between the hierarchical property of prescriptions and the molecular network correlations of the herb ingredients in prescriptions. We use entropy to measure the hierarchical organizing degree of a given prescription in terms of the distribution of herbal combinations.

After we obtained the herbal combinations of each prescription and all the prescription groups in our study (i.e., four types of clinical prescriptions), we could record each herbal combination with its weight and compute the distribution of the weight of herbal combinations for each prescription with regard to the global distribution of herbal combinations of each type of clinical prescriptions. Specifically, according to the weight distribution of herbal combinations of a given clinical prescription dataset, we divided the weight range (e.g., from 1 to 3,000) into four intervals of some specific value (i.e., 300 in our study). Then using the list of herbal combinations and their weights, we could further calculate the probability of herbal combinations belonging to each interval of the corresponding prescription group for a given prescription. Next, we quantified the hierarchical degree of a prescription based on the entropy measure (Shannon, 1948; Wallace et al., 2019) as follows (Eq. 4). The larger the entropy value, the stronger the hierarchical organization degree for a given prescription, which would medically mean a more systemically organized herbal prescription with hierarchical roles (i.e., tend to prescribe herbal combinations in one formula with diverse global weight ranges in each group). The higher the entropy value, the more the hierarchical organization of the prescription in terms of diverse roles.

$$E=\;-\sum_{i=1}^{n}p_{i}\log p_{i}$$ (4)

where n represents the number of weight intervals and is set to four in our analysis and $p_{i}$ represents the probability of the herbal combinations in each interval and is calculated as follows (Eq. 5):

$$p_{i}=\frac{N_{i}}{\sum_{i=1}^{n}N_{i}}$$ (5)

in which $N_{i}$ represents the number of herbal combinations in weight interval i.

Therefore, we could calculate entropy for all prescriptions for each type of the clinical prescriptions. Next, for each type of the clinical prescriptions, we calculate the Pearson Correlations Coefficient (PCC) between its entropy value and $S_{p}$ score to evaluate the statistical correlation between hierarchical degree and molecular network interactions of the herbal combinations for each prescription group.

## Results

### Herbal Combination Networks

According to the clinical formulae-herb relationships in outpatient, type 2 diabetes, CHD, qi deficiency, and blood stasis, we constructed five types of herbal combination networks in which nodes represented the herbs and edges represented the cooccurrence of herbs in clinical formulae. These five HCNets were described in brief in Table 2.

**TABLE 2:** The basic information of five different HCNets used for analysis.

	Outpatient	Diabetes	CHD	Blood stasis	Qi deficiency
Total number of nodes	576	492	436	439	422
Total number of edges	120,619	34,824	29,381	19,323	18,186
Average degree in network	419.82	142.56	135.78	89.64	87.39
Edge density	0.73	0.29	0.31	0.20	0.21
Average clustering coefficient	0.85	0.61	0.63	0.51	0.51

### Hierarchical Organization of Herbal Combinations in Clinical Prescriptions

**FIGURE 3:**
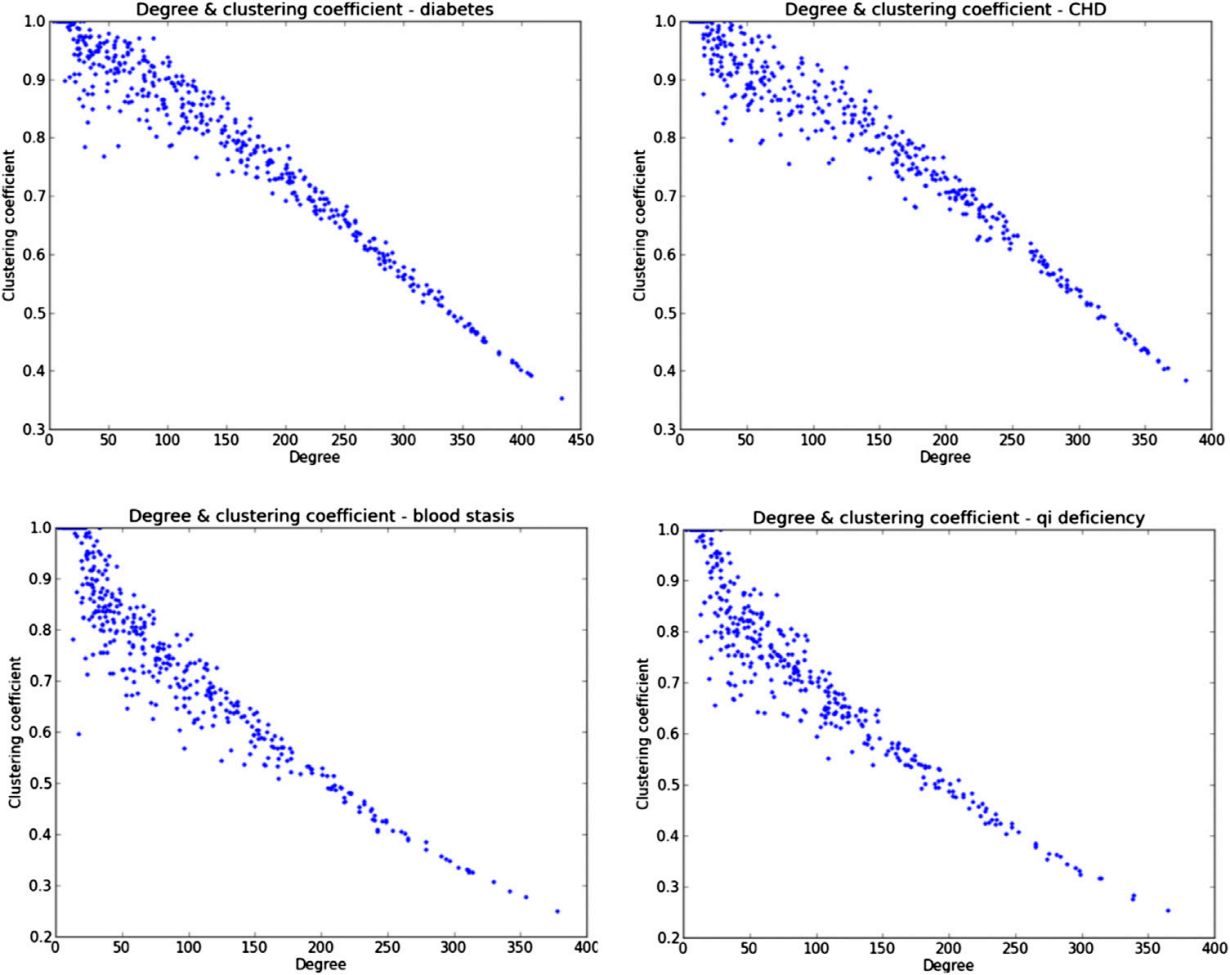
The relationship between clustering coefficient and node degree in HCNets. It indicated a clear linear negative correlation between the clustering coefficients and the degree of nodes.

To investigate the hierarchical properties of herbal combination networks, we evaluate the relationship between the clustering coefficient and node degree in the network. We performed experiments on four types of clinical prescriptions, and the results were shown in Figure 3.

We found that it displays a clear linear negative correlation between the clustering coefficients and the degree of nodes. It was found that, in real networks, clustering coefficients decrease with the vertex degree, which has been taken as a signature of the network hierarchical structure (Soffer and Vazquez, 2005). Therefore, this indicates that hierarchical properties exist in the herbal combination networks. Next, we would further investigate the scaling properties of HCNet according to node degree and weight distribution.

### Scaling Heterogeneity of Degree and Weight Distribution

**FIGURE 4:**
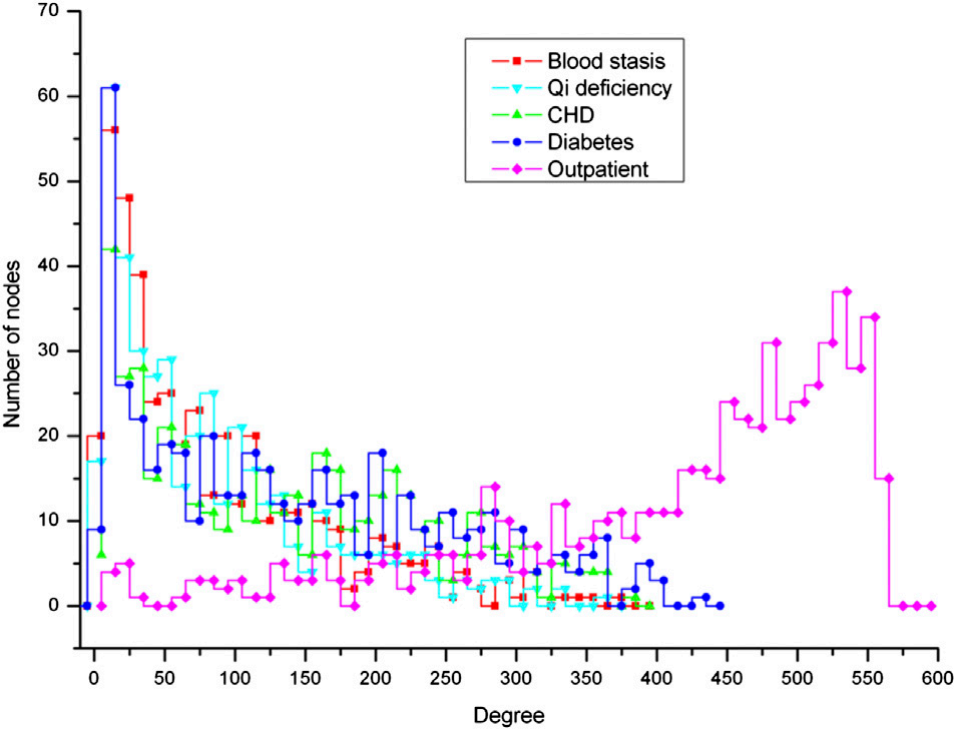
The degree distribution of herb in five HCNets. Four HCNets, namely, blood stasis, qi deficiency, CHD, and diabetes, have a similar distribution. However, the degree of outpatient HCNet obviously has a different distribution, of which most nodes have very high degrees (e.g., 500 more connections) while few nodes have low degrees (e.g., less than 20). In contrast to the real data, the node degrees in the networks derived from randomly shuffled data have a Poisson distribution for qi deficiency and blood stasis datasets, which have comparatively less clinical prescription samples.

To investigate the scaling properties of HCNet, we evaluate the node degree distribution and link weight distribution in the network. It showed that herbal combinations in clinical formulae are obeying a scaling property, which has significantly departed from Poisson distribution (Figure 4). Although the node degree distribution in HCNet is not power law (this is obviously rational because the capacity of herb dictionary is fixed and thus increase of nodes is not met in HCNet), it showed a significant heterogeneous scaling for different nodes. The degrees of herb nodes scale from less than ten (e.g., 5) to several hundred (e.g., 550), whose range is much wider than the corresponding Poisson distribution.

**FIGURE 5:**
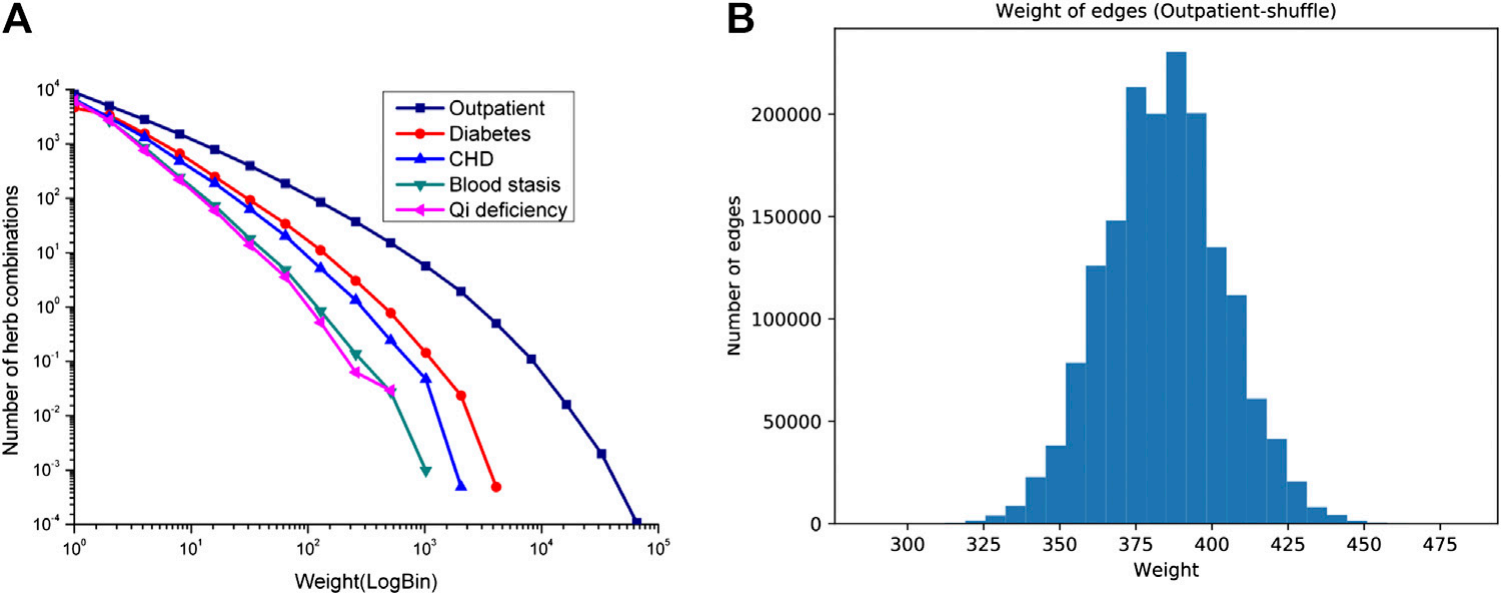
Link weight distributions in HCNets. **(A)** The empirical distribution of five clinical datasets. **(B)** The distribution of random permutated samples of outpatient dataset. The empirical distributions of five real-world clinical formulae display mainly a power law. The weights of links scale from one to tens of thousands that form a wide range. However, the link weight of the corresponding random dataset of outpatient case obeys Poisson distribution with a mean weight, not more than 400. In addition, we also analyzed the random cases of the other four diseases (Supplementary Figure S1), the link weight of the corresponding random dataset of diabetes and CHD case obeys Poisson distribution with mean weight not more than 15, and the link weight of the corresponding random dataset of qi deficiency and blood stasis case obeys seminormal distribution with a narrow weight scale.

Furthermore, it is interesting that the link weight distribution of HCNet is a clear power law, in which several herbal combinations are prescribed in high frequency while most of the other herbal combinations have very low frequency (Figure 5). This means that, in clinical practice, the prescription of herbal combinations for treatment is nonrandomly chosen and preferentially used by TCM physicians for the disease management of individualized patients.

We find that herbal combinations in four clinical formulae are obeying a scaling property (Figure 5), and it also means that the distribution of herbal combination has a hierarchical property. Next, we will calculate the degree of interaction between the targets in the context molecular network in herbal combinations and investigate whether frequently used herbal combinations hold close pharmacological effects.

### Molecular Network Interaction Patterns of Herbal Combinations

To evaluate the underlying pharmacological mechanisms of herbal combinations in clinical prescriptions, we quantified the network correlations between the targets of the herbal ingredients of the four types of clinical prescriptions for diabetes, CHD, qi deficiency, and blood stasis (see Methods section, *Network-Based Measures of Molecular Interactions Among Herbs in Prescriptions*).

We collected the protein targets of herbs in prescriptions from HIT database (Ye et al., 2011), which is a comprehensive and fully curated database of herbs and their corresponding protein targets. Due to the lack of target information for some herbs according to HIT database, at the same time, to assure each prescription should contain at least two herbs in the analysis. Finally, we quantified the molecular network correlations of those herbs in 21,566, 9,051, 2,392, and 2,801 prescriptions from diabetes, CHD, qi deficiency, and blood stasis, respectively, in which the number of herbs containing known targets is greater than or equal to two. The mean and standard deviation (std) of $S_{p}$ scores of four types of prescriptions were shown in Table 3.

**TABLE 3:** The mean and standard deviation of $S_{p}$ scores of four types of clinical prescriptions and TEFD.

Type	Diabetes	CHD	Qi deficiency	Blood stasis	TEFD	Random
Mean	−0.03	−0.05	−0.09	−0.11	−0.09	0.16
Std	0.32	0.33	0.28	0.28	0.15	0.08

In order to give a benchmark reference for the four types of clinical prescriptions, we selected the classical prescriptions of TCM, Treatise on Exogenous Febrile Disease (TEFD), for comparative experiments. The TEFD contained 113 classical prescriptions in total, of which 85 prescriptions with associated targets were selected for quantification. The number of herbs containing known targets is greater than or equal to two in 85 prescriptions. We collected the classical prescriptions from TEFD as a representative prescription benchmark with high-quality herbal combinations because most prescriptions in TEFD have been widely used in TCM clinical practice for thousands of years (Li et al., 2010a; Wang and Xiao, 2010; Zhang et al., 2010). Therefore, in general, we will consider that the herbal combinations held in the prescriptions from TEFD give a better example than those from the above four types of clinical prescriptions.

**FIGURE 6:**
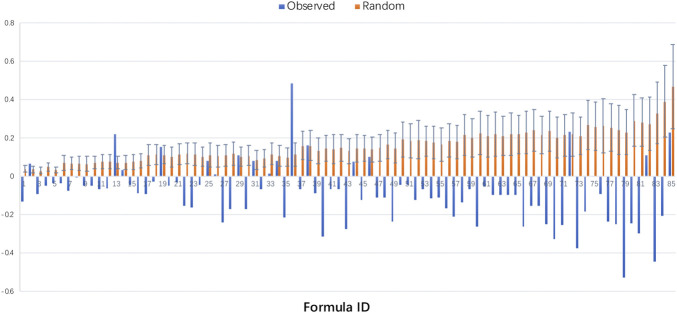
The $S_{p}$ scores of prescriptions in Treatise on Exogenous Febrile Disease. We ranked the prescriptions in descending order according to the number of herbs and compared the $S_{p}$ score distribution between prescriptions in TEFD and random prescriptions. The $S_{p}$ scores of prescriptions in TEFD were shown as blue columnar and random prescriptions were shown as orange columnar.

Next, we quantified the degree of interactions between the herbal combinations in 85 prescriptions from TEFD in the context of the human PPI network (Figure 6). For comparison of random expectations, we reshuffled (1,000 random permutations) the herbs in each prescription by using the Fisher-Yates method (Fisher and Yates, 1948). Finally, we found that the $S_{p}$ scores of prescriptions in TEFD were significantly lower than those of random prescriptions (*p* = 2.46718e-27). We found that 92% of real prescriptions in TEFD had lower $S_{p}$ scores compared to random prescriptions, and $S_{p}$ scores of 79% of real prescriptions were less than 0, but $S_{p}$ scores of all random prescriptions were greater than 0. And we found that the mean $S_{p}$ score in TEFD was -0.09, but the random was 0.16, indicating that the target modules of herbs in a prescription were closely related to each other in the context of the molecular network. To further evaluate the quality of herbal combinations of current clinical prescriptions, with similar calculation methods, we obtained the $S_{p}$ scores of each prescription in the four clinical prescriptions datasets. The results showed that the mean of $S_{p}$ scores of all four clinical prescriptions was less than 0. This means that most real-world clinical prescriptions would tend to have a high degree of herbal combinations as good as those of TEFD prescriptions. It is practical and reasonable that, in TCM clinical settings, most practitioners would directly adopt the classical prescriptions or use the classical prescriptions as a basic therapeutic framework for clinical treatments (Duan et al., 2018; Chen et al., 2019).

### Molecular Correlations of Hierarchical Herbal Combinations in Clinical Prescriptions

We have found that the weights of herbal combinations scale from one to tens of thousands that form a wide range and the distribution of herbal combinations has a hierarchical organization property in our previous analysis. Meanwhile, we also obtained the molecular measures of herbal combinations in prescriptions in terms of their molecular interactions. Next, we would expect the potential associations between these two indexes for a given prescription (see Methods section, *Hierarchical Organization Measure of Clinical Prescriptions*).

**FIGURE 7:**
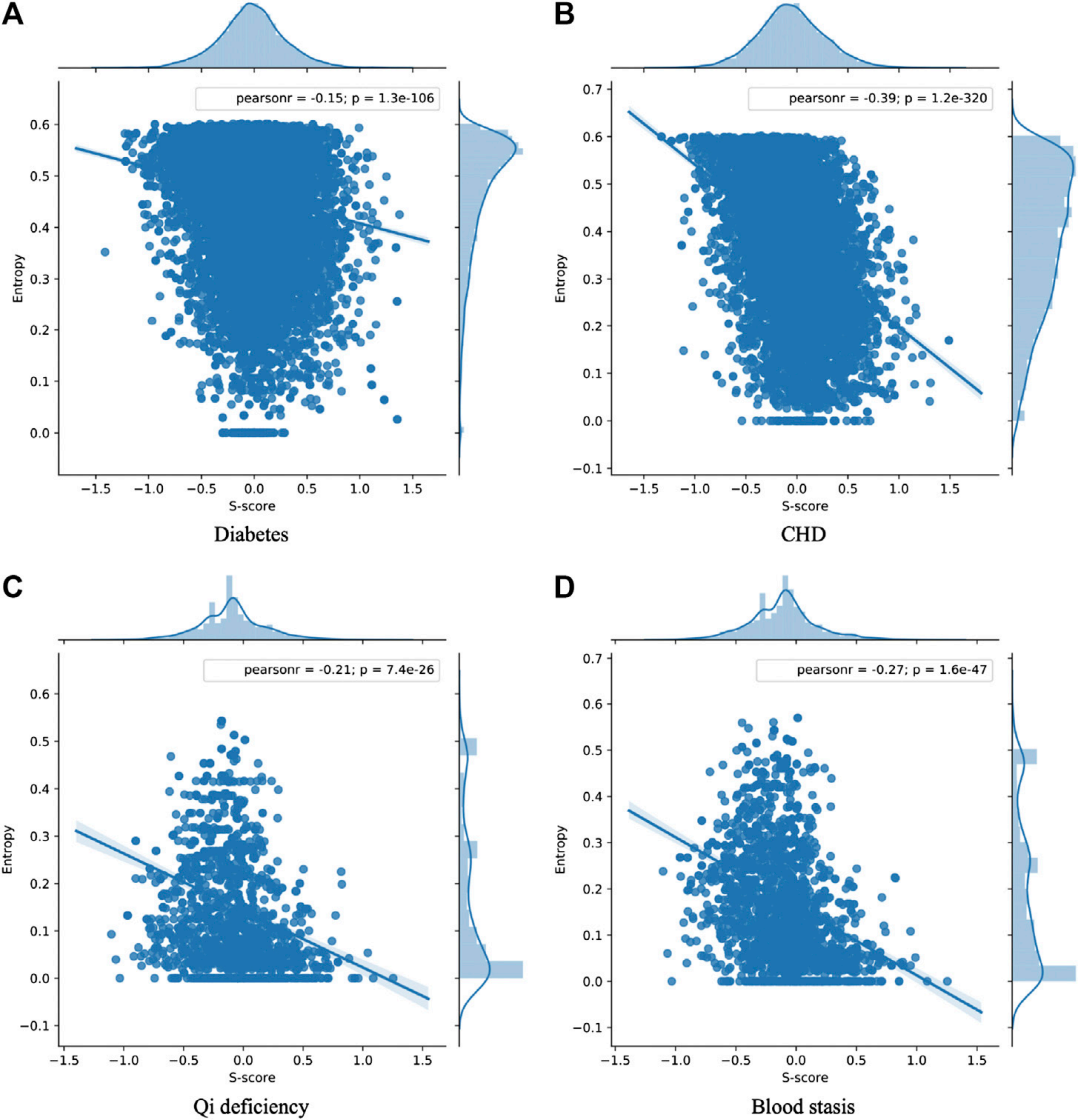
The Pearson correlation coefficient for four types of clinical prescriptions. We could find that entropy value and S score of diabetes **(A)**, CHD **(B)**, qi deficiency **(C),** and blood stasis **(D)** were negatively correlated.

To test the assumption, we performed a Pearson correlation analysis on its entropy value and $S_{p}$ score. The PCC and *p*-value were shown in Figure 7. We could find that entropy value and $S_{p}$ score of clinical prescriptions were negatively correlated with PCC ranging from −0.15 to −0.39 (Figure 7A, p = 1.3e−106; Figure 7B,  p = 1.2e−320; Figure 7C,  p = 7.4e−26; Figure 7D,  p = 1.6e−47) for the four clinical herbal prescriptions. This indicated that the larger the entropy value of a given prescription, the smaller the $S_{p}$ score of the prescription. Therefore, it indicated that the more the hierarchical herbal combinations were involved in a prescription, the more the connected molecular networks would be found between the targets of the herbal ingredients in a given prescription. The results also reflected exactly a well-recognized principle called JCZS in TCM formula theories, which illustrates that a well-organized formula should consist of multiple herbal ingredients with differentiated therapeutic roles for targeted disease conditions.

## Discussion and Conclusion

TCM herbal prescription which usually includes tens of different herbal ingredients as a single therapeutic formula is the main kind of intervention for chronic disease treatment in China (Qi et al., 2013). Typically, it is a kind of combination therapies with complicated underlying molecular pharmacological mechanisms (Chan et al., 2012). Combination therapies offer promising therapeutic solutions for complex disease treatments, from hypertension, COPD, and cancer with potential high efficacies and lower adverse effects (Yki-Jarvinen, 2001; Margulies and Hicks, 2009). However, the clinical organization principle and its underlying network mechanisms still need to be elucidated. TCM herbal prescription has been widely used as a combination therapy in clinical settings (He et al., 2012; Liu et al., 2013; Duan et al., 2018) by adhering to empirical clinical regularities, such as JCZS (He et al., 2015; Duan et al., 2018), and frequently used herbal combinations. Connecting those clinical regularities with the molecular network patterns related to the targets of herbal ingredients would potentially improve the pharmacological understanding of the empirical rules to develop more effective combination therapies.

**FIGURE 8:**
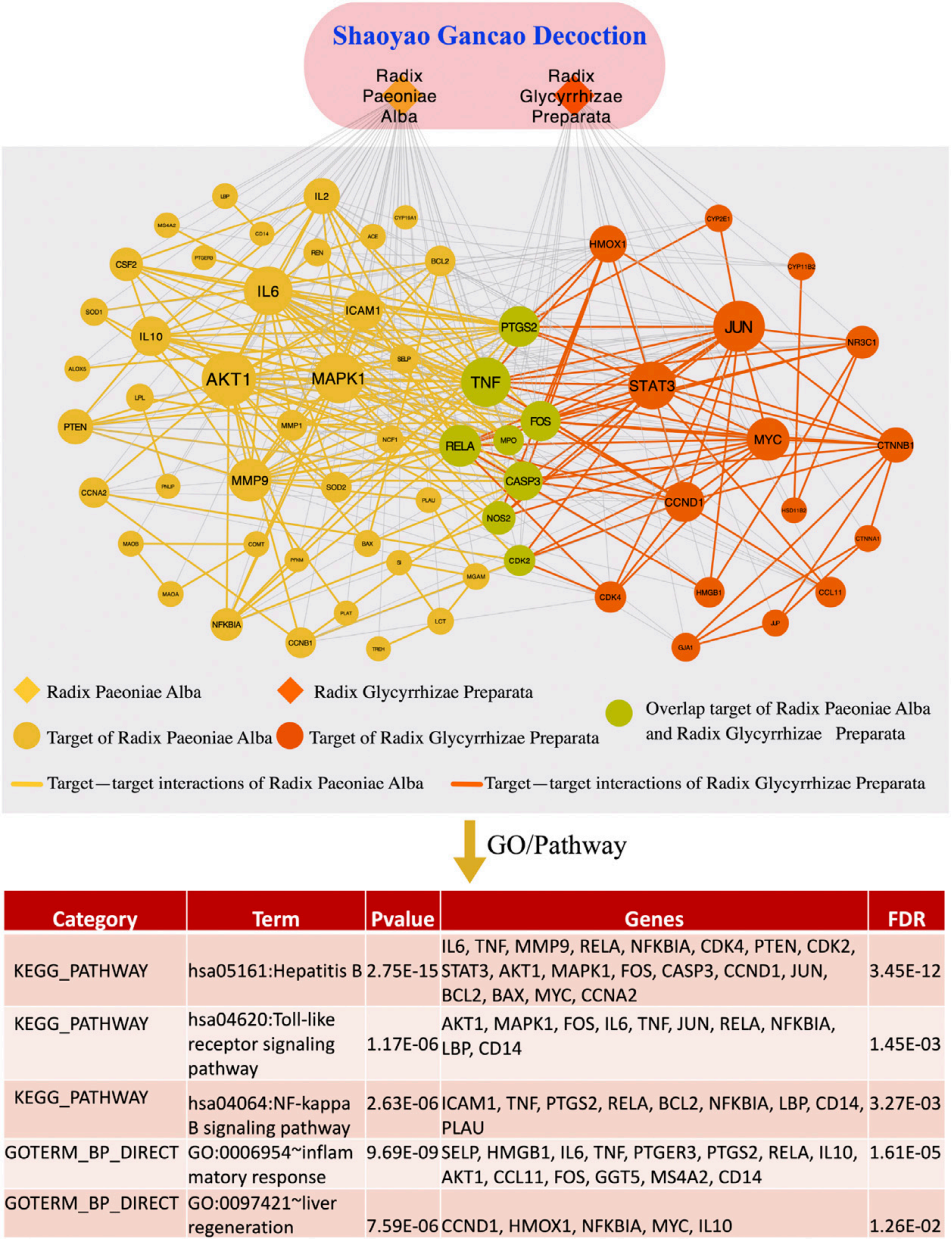
The network molecular associations for the only two herbs, namely, Radix Paeoniae Alba and Radix Glycyrrhizae Preparata in the Shaoyao Gancao decoction, (SGD). **(A)** Target modules of the two herbs and their interactions. Yellow nodes represent the targets of Radix Paeoniae Alba, and red nodes represent the targets of Radix Glycyrrhizae Preparata, while the edges represent the interactions between the targets in the PPI network. Nodes with green color represent overlapping targets of two herbs. **(B)** The related gene ontology and pathways of target modules of the two herbs.

The well-recognized principle called JCZS in TCM formula theories means that a well-organized formula should consist of multiple herbal ingredients with different therapeutic roles (Wang et al., 2008). This would imply the existence of a hierarchical organization of herbal combinations and the underlying network mechanisms of herbal ingredients for a well-established set of clinical prescriptions (e.g., the herbal prescriptions in TEFD). Here, we investigated the clinical regularities of herbal combinations involved in each prescription in terms of network topological patterns and applied a network-based measurement (Cheng et al., 2019) to quantify the molecular network interactions between herb target modules in a given herbal prescription. Our results indicate that there do exist clinical prescription rules to choose herbal combinations in a clinical prescription, which can be displayed by the scaling and hierarchical topological properties of the herbal combination network. Specifically, a clinical prescription would tend to consist of both highly used herbs or herbal combinations and less used herbs or herbal combinations as a whole formula for disease treatment. This might resemble to the well-established prescription principle (i.e., JCZS) for TCM herbal prescriptions, which implies a good prescription need include herbs with different pharmacological effects. Furthermore, we found that the network-based measure of high-quality drug combinations (Cheng et al., 2019) could be used for evaluating the molecular interaction closeness of both herbal combinations and prescriptions. For example, a classical formula in TEFD, “Shaoyao Gancao decoction” (SGD), contains two herbs: Radix Paeoniae Alba (hRPA) and Radix Glycyrrhizae Preparata (hRGP), which has been further prescribed as core herbal combinations in many classical formulae [e.g., Mahuang Shengma decoction (Fan et al., 2011), Xiaoqinglong decoction (Wang et al., 2018), Gegen decoction (Chai et al., 2020), Chaihu Guizhi decoction (Li et al., 2019), and HuangQin decoction (Dai et al., 2018)]. According to the molecular network-based measurement, we found that the $S_{p}$ score of SGD was -0.03 (the random expectation was 0.03). This means that close interactions are involved between the target modules of the two-member herbs in SGD. Actually, by identifying the herb-target associations from HIT database, we found that there are 47 and 24 potential targets of hRPA and hRGP, respectively. These two target sets each form a rather dense PPI subnetwork (we call it a module, Figure 8A). There are eight common targets shared by hRPA and hRGP, such as TNF, CASP3, RELA, and NOS2. Meanwhile, 75 interactions exist between the target modules of hRPA and hRGP (with 0.113 network density). We further used DAVID (Huang Da et al., 2009) to obtain the enriched GO and KEGG pathways (Figure 8B). The results showed that SGD enriched the inflammation-related pathways such as Hepatitis B, NF-kB, and TLRs (Mantovani, 2010) and liver-related GO annotations such as liver regeneration, which could partially explain the clinical effects of SGD for treating liver disorders and anti-inflammatory in clinic settings (Bi et al., 2014).

Overall, we have been working to investigate the network topological regularities and the underlying molecular network mechanisms of herbal prescriptions, and we hope that our work and findings for clinical prescriptions would give references for understanding of the principles and molecular mechanisms of multidrug combinations (Dawson and Carragher, 2014; Cheng et al., 2019; Zhang et al., 2019) after fully digging the hidden knowledge underlying the effective multiple therapies (e.g., Chinese herbal prescriptions).

This study has several limitations. First, since it is not aiming to predict the specific knowledge on pharmacological effects of specific prescriptions or drugs, unlike the study on pharmacological research of a given herbal prescription or predicting a novel target, it would be difficult for us to design a straightforward wet-lab experiment to validate the rationality of organizing herb combinations in a good clinical prescription. However, large-scale wet-lab experiments on validating the differentiated molecular patterns between high frequently used herbal combinations and rarely used herbal combinations should be designed and performed in further research to improve the reliability of the network analysis results. Second, currently, we only used HIT database as the herb-target associations for molecular network-related analysis, which would limit the number of herb-target associations and thus influence the generalization of our analysis. We would integrate other well-established network pharmacological databases, such as ETCM (Xu et al., 2019) and SymMap (Wu et al., 2019) to further validate the underlying molecular network regularities of herbal prescriptions. Third, due to the complexity of clinical conditions of individualized patients, it is important that the prescribed herbal treatments for specific patients should be evaluated by the outcomes and finally help figure out the true high-quality clinical herbal prescriptions from a large-scale clinical dataset. Therefore, we would extend the evaluation measure together with disease conditions to find good herbal prescriptions with potential effectiveness. However, without the related in vitro or in vivo experimental validations, we believe that our current research could deliver certain potential insights for the field of network pharmacology and combination drug development. By evaluating the underlying molecular network patterns of the herbal combinations in herbal prescriptions, it would be promising to propose a novel network pharmacological approach to investigate the inherent network mechanisms (e.g., organizing of herbal combinations) of a given clinical prescription and thus help design combination therapies with better effectiveness for disease treatment.

## Data Availability Statement

All datasets presented in this study are included in the article/Supplementary Material.

## Author Contributions

XZ, ZG, RZ, and BL conceived and designed the study. NW and ND analyzed data and wrote the draft manuscript; KY, ZS, and KC collected related data; YP, RZ, DW, JY, CJ, YZ, and XL reviewed the methods and the results. All authors have reviewed the manuscript; in particular, XZ and DW have revised the manuscript with the help of other coauthors.

## Funding

The work is supported by the National Key Research and Development Program (2017YFC1703506), the Special Programs of Traditional Chinese Medicine (201407001, JDZX2015170, and JDZX2015171), National Major Scientific and Technological Special Project for "Significant New Drugs Development" (2019ZX09201005-002-006) and the National Key Technology R&D Program (2013BAI02B01 and 2013BAI13B04).

## Conflict of Interest

The authors declare that the research was conducted in the absence of any commercial or financial relationships that could be construed as a potential conflict of interest.

## References

[B1] Aagaard L. Hansen E. H. (2013). Adverse drug reactions reported by consumers for nervous system medications in Europe 2007 to 2011. BMC Pharmacol. Toxicol. 14, 30 10.1186/2050-6511-14-30 23763896 PMC3685574

[B2] Ascierto P. A. Marincola F. M. (2011). Combination therapy: the next opportunity and challenge of medicine. J. Transl. Med. 9, 115 10.1186/1479-5876-9-115 21777474 PMC3157452

[B3] Bi X. Gong M. Di L. (2014). Review on prescription compatibility of shaoyao gancao decoction and reflection on pharmacokinetic compatibility mechanism of traditional Chinese medicine prescription based on *in vivo* drug interaction of main efficacious components. Evid. Based Complement Alternat. Med. 2014, 208129 10.1155/2014/208129 25147573 PMC4132488

[B4] Bozic I. Reiter J. G. Allen B. Antal T. Chatterjee K. Shah P. (2013). Evolutionary dynamics of cancer in response to targeted combination therapy. Elife. 2, e00747 10.7554/eLife.00747 23805382 PMC3691570

[B5] Chai C. Hong F. Yan Y. Yang L. Zong H. Wang C. (2020). Effect of traditional Chinese medicine formula GeGen decoction on primary dysmenorrhea: a randomized controlled trial study. J. Ethnopharmacol. 261, 113053 10.1016/j.jep.2020.113053 32534120

[B6] Chan K. Shaw D. Simmonds M. S. Leon C. J. Xu Q. Lu A. (2012). Good practice in reviewing and publishing studies on herbal medicine, with special emphasis on traditional Chinese medicine and Chinese materia medica. J. Ethnopharmacol. 140, 469–475. 10.1016/j.jep.2012.01.038 22330011

[B7] Chen Z. K. Wang X. N. Li Y. Y. Wang Y. H. Tang K. L. Wu D. F. (2019). Comparative network pharmacology analysis of classical TCM prescriptions for chronic liver disease. Front. Pharmacol. 10, 1353 10.3389/fphar.2019.01353 31824313 PMC6884058

[B8] Cheng F. X. Kovacs I. A. Barabasi A. L. (2019). Network-based prediction of drug combinations. Nat. Commun. 10, 1197 10.1038/s41467-019-09186-x 30867426 PMC6416394

[B9] Dai X. M. Cui D. N. Wang J. Zhang W. Zhang Z. J. Xu F. G. (2018). Systems pharmacology based strategy for Q-markers discovery of HuangQin decoction to attenuate intestinal damage. Front. Pharmacol. 9, 236 10.3389/fphar.2018.00236 29615909 PMC5870050

[B10] Dawson J. C. Carragher N. O. (2014). Quantitative phenotypic and pathway profiling guides rational drug combination strategies. Front. Pharmacol. 5, 118 10.3389/fphar.2014.00118 24904421 PMC4035564

[B11] Duan D. D. Wang Z. Wang Y. Y. (2018). New omic and network paradigms for deep understanding of therapeutic mechanisms for Fangji of traditional Chinese medicine. Acta Pharmacol. Sin. 39, 903–905. 10.1038/aps.2018.42 29863110 PMC6256266

[B12] Duke J. Friedlin J. Ryan P. (2011). A quantitative analysis of adverse events and “overwarning” in drug labeling. Arch. Intern. Med. 171, 944–946. 10.1001/archinternmed.2011.182 21606101

[B13] Fan F. J. Hong W. X. Li X. Zhang T. Liu X. L. Chen W. D. (2011). “Research on compatibility of prescription of TCP based on the principle of attribute partial order chart,” in Proceedings of the 2011 first international conference on instrumentation, measurement, computer, communication and control, Beijing, China, October 21–23, 2011, 82–86. 10.1109/IMCCC.2011.30

[B14] Fisher R. A. Yates F. (1948). Statistical tables for biological, agricultural and medical research. London, United Kingdom: Oliver & Boyd.

[B15] He Y. H. Zheng X. Sit C. Loo W. T. Wang Z. Y. Xie T. (2012). Using association rules mining to explore pattern of Chinese medicinal formulae (prescription) in treating and preventing breast cancer recurrence and metastasis. J. Transl. Med. 10, S12 10.1186/1479-5876-10-S1-S12 23046537 PMC3445862

[B16] He B. Lu C. Wang M. L. Zheng G. Chen G. Jiang M. (2015). Drug discovery in traditional Chinese medicine: from herbal fufang to combinatory drugs. Science. 350, S74–S76.

[B17] He D. Huang J. H. Zhang Z. Y. Du Q. Peng W. J. Yu R. (2019). A network pharmacology-based strategy for predicting active ingredients and potential targets of LiuWei DiHuang pill in treating type 2 diabetes mellitus. Drug Des. Dev. Ther. 13, 3989–4005. 10.2147/Dddt.S216644 PMC6890936 31819371

[B18] Huang Da W. Sherman B. T. Lempicki R. A. (2009). Bioinformatics enrichment tools: paths toward the comprehensive functional analysis of large gene lists. Nucleic Acids Res. 37, 1–13. 10.1093/nar/gkn923 19033363 PMC2615629

[B19] Lasser K. E. Allen P. D. Woolhandler S. J. Himmelstein D. U. Wolfe S. M. Bor D. H. (2002). Timing of new black box warnings and withdrawals for prescription medications. J. Am. Med. Assoc. 287, 2215–2220. 10.1001/jama.287.17.2215 11980521

[B20] Lazarou J. Pomeranz B. H. Corey P. N. (1998). Incidence of adverse drug reactions in hospitalized patients: a meta-analysis of prospective studies. J. Am. Med. Assoc. 279, 1200–1205. 10.1001/jama.279.15.1200 9555760

[B21] Li J. Liu B. X. Zhang S. G. Ma H., X. Zhang Q. N. Lu Z. Y. (2010a). Study on effective powers of familiar herbs in treatise on exogenous febrile disease of shanghan lun. J. of Liaoning Univ. of Trad. Chinese Med. 02, 47–48. 10.13194/j.jlunivtcm.2010.02.49.lij.023

[B22] Li S. Zhang B. Jiang D. Wei Y. Y. Zhang N. B. (2010b). Herb network construction and co-module analysis for uncovering the combination rule of traditional Chinese herbal formulae. BMC Bioinf. 11, S6 10.1186/1471-2105-11-S11-S6 PMC3024874 21172056

[B23] Li Z. Wen R. Du Y. Zhao S. Zhao P. Jiang H. (2019). Simultaneous quantification of fifteen compounds in rat plasma by LC-MS/MS and its application to a pharmacokinetic study of Chaihu-Guizhi decoction. J. Chromatogr. B Analyt. Technol. Biomed. Life Sci. 1105, 15–25. 10.1016/j.jchromb.2018.12.006 30554094

[B24] Liu Z. Chen S. Cai J. Zhang E. Lan L. Zheng J. (2013). Traditional Chinese medicine syndrome-related herbal prescriptions in treatment of malignant tumors. J. Tradit. Chin. Med. 33, 19–26. 10.1016/s0254-6272(13)60095-3 23596807

[B25] Luzuriaga K. Bryson Y. Krogstad P. Robinson J. Stechenberg B. Lamson M. (1997). Combination treatment with zidovudine, didanosine, and nevirapine in infants with human immunodeficiency virus type 1 infection. N. Engl. J. Med. 336, 1343–1349. 10.1056/NEJM199705083361902 9134874

[B26] Mantovani A. (2010). Molecular pathways linking inflammation and cancer. Curr. Mol. Med. 10, 369–373. 10.2174/156652410791316968 20455855

[B27] Margulies S. Hicks R. (2009). Combination therapies for traumatic brain injury: prospective considerations. J. Neurotrauma. 26, 925–939. 10.1089/neu.2008-0794 19331514 PMC2857809

[B28] Menche J. Sharma A. Kitsak M. Ghiassian S. D. Vidal M. Loscalzo J. (2015). Disease networks. Uncovering disease-disease relationships through the incomplete interactome. Science. 347, 1257601 10.1126/science.1257601 25700523 PMC4435741

[B29] Mottonen T. Hannonen P. Leirisalo-Repo M. Nissila M. Kautiainen H. Korpela M. (1999). Comparison of combination therapy with single-drug therapy in early rheumatoid arthritis: a randomised trial. FIN-RACo trial group. Lancet. 353, 1568–1573. 10.1016/s0140-6736(98)08513-4 10334255

[B30] Pritchard J. R. Bruno P. M. Gilbert L. A. Capron K. L. Lauffenburger D. A. Hemann M. T. (2013). Defining principles of combination drug mechanisms of action. Proc. Natl. Acad. Sci. U.S.A. 110, E170–E179. 10.1073/pnas.1210419110 23251029 PMC3545813

[B31] Qi F. H. Wang Z. X. Cai P. P. Zhao L. Gao J. J. Kokudo N. (2013). Traditional Chinese medicine and related active compounds: a review of their role on hepatitis B virus infection. Drug Discov Ther. 7, 212–224. 10.5582/ddt.2013.v7.6.212 24423652

[B32] Routledge P. A. O’mahony M. S. Woodhouse K. W. (2004). Adverse drug reactions in elderly patients. Br. J. Clin. Pharmacol. 57, 121–126. 10.1046/j.1365-2125.2003.01875.x 14748810 PMC1884428

[B33] Shannon C. E. (1948). A mathematical theory of communication. Bell Syst. Tech. 27, 379–423. 10.1002/j.1538-7305.1948.tb01338.x

[B34] Smyth R. M. Gargon E. Kirkham J. Cresswell L. Golder S. Smyth R. (2012). Adverse drug reactions in children--a systematic review. PLoS One. 7, e24061 10.1371/journal.pone.0024061 22403604 PMC3293884

[B35] Soffer S. N. Vazquez A. (2005). Network clustering coefficient without degree-correlation biases. Phys. Rev. E—Stat. Nonlinear Soft Matter Phys. 71, 057101 10.1103/PhysRevE.71.057101 16089694

[B36] Wald D. S. Law M. Morris J. K. Bestwick J. P. Wald N. J. (2009). Combination therapy versus monotherapy in reducing blood pressure: meta-analysis on 11,000 participants from 42 trials. Am. J. Med. 122, 290–300. 10.1016/j.amjmed.2008.09.038 19272490

[B37] Wallace Z. S. Rosenthal S. B. Fisch K. M. Ideker T. Sasik R. (2019). On entropy and information in gene interaction networks. Bioinformatics. 35, 815–822. 10.1093/bioinformatics/bty691 30102349 PMC7245360

[B38] Wang Z. L. Xiao X. R. (2010). Relationship between the amount of water added and the weight of drugs for decoction in Treatise on exogenous febrile diseases. Liaoning J. of Trad. Chinese Med. 3, 433–435. 10.13192/j.ljtcm.2010.03.54.wangzhl.019

[B39] Wang L. Zhou G. B. Liu P. Song J. H. Liang Y. Yan X. J. (2008). Dissection of mechanisms of Chinese medicinal formula Realgar-Indigo naturalis as an effective treatment for promyelocytic leukemia. Proc. Natl. Acad. Sci. U.S.A. 105, 4826–4831. 10.1073/pnas.0712365105 18344322 PMC2290784

[B40] Wang Q. Yao G. Z. Pan G. M. Huang J. Y. An Y. P. Zou X. (2017). [Analysis of on medication rules for Qi-deficiency and blood-stasis syndrome of chronic heart failure based on data mining technology]. Zhongguo Zhongyao Zazhi. 42, 182–186. 10.19540/j.cnki.cjcmm.20161222.040 28945046

[B41] Wang H. Mao B. Chen C. (2018). Xiaoqinglong decoction attenuates chronic obstructive pulmonary disease in rats via inhibition of autophagy. Evid. Based Complement. Alternat. Med. 2018, 6705871 10.1155/2018/6705871 29636783 PMC5831972

[B42] Wu Y. Zhang F. Yang K. Fang S. Bu D. Li H. (2019). SymMap: an integrative database of traditional Chinese medicine enhanced by symptom mapping. Nucleic Acids Res. 47, D1110–D1117. 10.1093/nar/gky1021 30380087 PMC6323958

[B43] Xu H. Y. Zhang Y. Q. Liu Z. M. Chen T. Lv C. Y. Tang S. H. (2019). ETCM: an encyclopaedia of traditional Chinese medicine. Nucleic Acids Res. 47, D976–D982. 10.1093/nar/gky987 30365030 PMC6323948

[B44] Yardley D. A. (2013). Drug resistance and the role of combination chemotherapy in improving patient outcomes. Int J Breast Cancer. 2013, 137414 10.1155/2013/137414 23864953 PMC3707274

[B45] Ye H. Ye L. Kang H. Zhang D. F. Tao L. Tang K. L. (2011). HIT: linking herbal active ingredients to targets. Nucleic Acids Res. 39, D1055–D1059. 10.1093/nar/gkq1165 21097881 PMC3013727

[B46] Yki-Jarvinen H. (2001). Combination therapies with insulin in type 2 diabetes. Diabetes Care. 24, 758–767. 10.2337/diacare.24.4.758 11315844

[B47] Zhang G. J. Liu E. S. Wang D. Q. Liu C. X. (2010). Discussing relevance of lung and large intestine from Treatise on exogenous febrile diseases. Tianjin Journal of Traditional Chinese Medicine. 4, 299–300.

[B48] Zhang M. Lee S. Yao B. Xiao G. Xu L. Xie Y. (2019). DIGREM: an integrated web-based platform for detecting effective multi-drug combinations. Bioinformatics. 35, 1792–1794. 10.1093/bioinformatics/bty860 30295728 PMC6513155

[B49] Zhou X. Z. Chen S. B. Liu B. Y. Zhang R. S. Wang Y. H. Li P. (2010). Development of traditional Chinese medicine clinical data warehouse for medical knowledge discovery and decision support. Artif. Intell. Med. 48, 139–152. 10.1016/j.artmed.2009.07.012 20122820

