# Supplementary material for: Network Patterns of Herbal Combinations in Traditional Chinese Clinical Prescriptions
Source: Front Pharmacol. 2021 Jan 20;11:590824. doi: 10.3389/fphar.2020.590824 (PMC7854460; doi:10.3389/fphar.2020.590824)
Supplement: Supplementary file 1 [file datasheet1.docx]

Supplementary Material

# Supplementary Figures and Tables

## Supplementary Figures


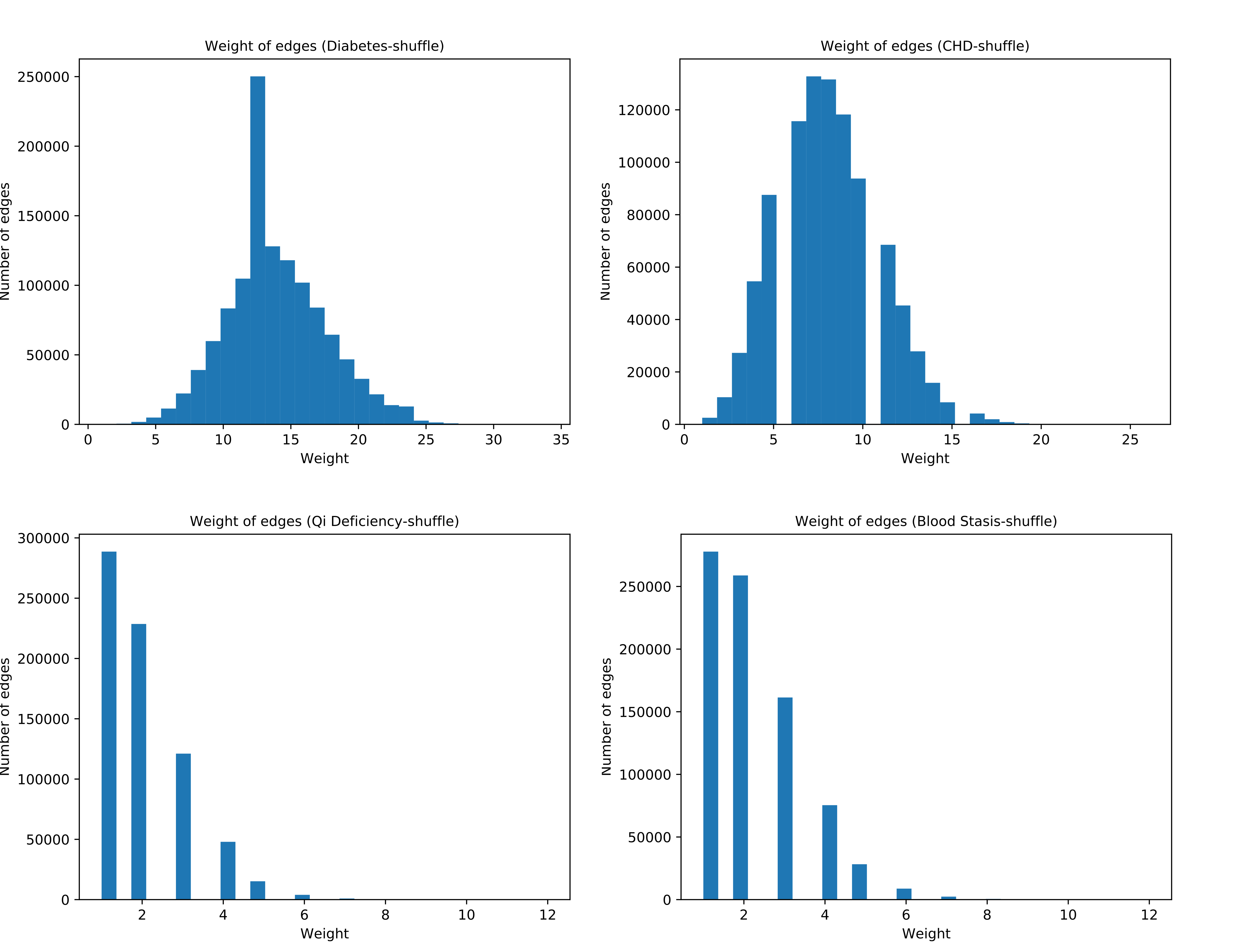


**Supplementary Figure 1.** Link weight distributions in random permutated samples of diabetes, CHD, qi deficiency and blood stasis. The link weight of the corresponding random data set of diabetes and CHD case obeys Possion distribution with mean weight not more than 15, and the link weight of the corresponding random data set of qi deficiency and blood stasis case obeys semi normal distribution with narrow weight scale.
